# Isoquinoline Alkaloids in Sows’ Diet Reduce Body Weight Loss during Lactation and Increase IgG in Colostrum

**DOI:** 10.3390/ani11082195

**Published:** 2021-07-23

**Authors:** Ester Arévalo Sureda, Xuemei Zhao, Valeria Artuso-Ponte, Sophie-Charlotte Wall, Bing Li, Wei Fang, Julie Uerlings, Yuping Zhang, Martine Schroyen, Clément Grelet, Frédéric Dehareng, José Wavreille, Nadia Everaert

**Affiliations:** 1 Precision Livestock and Nutrition Laboratory, TERRA Teaching and Research Centre, GeQAmbloux Agro-Bio Tech, University of Liège, 5030 Gembloux, Belgium; ester.arevalosureda@uliege.be (E.A.S.); zh_xm@aliyun.com (X.Z.); libing881011@163.com (B.L.); fangwei1987@126.com (W.F.); julie.uerlings@gmail.com (J.U.); zhangyuping@caas.cn (Y.Z.); Martine.Schroyen@uliege.be (M.S.); 2 Phytobiotics Futterzusatzstoffe GmbH, 65343 Eltville, Germany; v.artuso@phytobiotics.com (V.A.-P.); sc.wall@phytobiotics.com (S.-C.W.); 3 Valorisation of Agricultural Products Department, Walloon Agricultural Research Centre, 5030 Gembloux, Belgium; c.grelet@cra.wallonie.be (C.G.); f.dehareng@cra.wallonie.be (F.D.); 4 Production and Sectors Department, Walloon Agricultural Research Centre, 5030 Gembloux, Belgium; j.wavreille@cra.wallonie.be

**Keywords:** farrowing stress, maternal programming, piglets, colostrum, growth performance

## Abstract

**Simple Summary:**

Plant extracts containing isoquinoline alkaloids (IQ) have been demonstrated to have anti-inflammatory properties. In pigs, IQ supplementation has been shown to downregulate the stress response and improve the digestibility of nutrients. The present experiment was conducted to test the hypothesis that supplementing sows’ diets with IQ during gestation would decrease stress at farrowing and improve colostrum quality, positively affecting the piglets’ health and performance.

**Abstract:**

Isoquinoline alkaloids (IQ) exert beneficial antimicrobial and anti-inflammatory effects in livestock. Therefore, we hypothesized that supplementing sows’ diets with IQ during gestation would decrease farrowing stress, affecting the piglets’ development and performance. Sows were divided into: IQ1, supplemented with IQ from gestation day 80 (G80) to weaning; IQ2, supplemented from gestation day 110 (G110) to weaning, and a non-supplemented (NC) group. Sow body weight (BW), feed intake, back-fat thickness and back-muscle thickness were monitored. Cortisol, glucose and insulin were measured in sows’ blood collected 5 d before, during, and after 7 d farrowing. Protein, fat, IgA and IgG were analyzed in the colostrum and milk. Piglets were monitored for weight and diarrhea score, and for ileum histology and gene expression 5 d post-weaning. IQ-supplemented sows lost less BW during lactation. Glucose and insulin levels were lower in the IQ groups compared to NC-sows 5 d before farrowing and had higher levels of protein and IgG in their colostrum. No other differences were observed in sows, nor in the measured parameters in piglets. In conclusion, IQ supplementation affected sows’ metabolism, reducing body weight loss during lactation. Providing IQ to sows from their entrance into the maternity barn might be sufficient to induce these effects. IQ improved colostrum quality, increasing the protein and IgG content, improving passive immunity for piglets.

## 1. Introduction

Isoquinoline alkaloids (IQ) comprise sanguinarine, chelerythrine, protopine, and allocryptopine, among others, and can be obtained from the plant *Macleaya cordata,* or plume poppy. The extract of *Macleaya cordata* has been shown to be particularly rich in sanguinarine [1]. Isoquinoline alkaloids (IQ), e.g., sanguinarine, have low bioavailability due to poor water-solubility, nonetheless, it has been shown that they can be metabolized in the pig intestine into a more easily absorbable form, and both compounds were found in the plasma of orally supplemented pigs [2]. Nevertheless, a study using 100 mg/kg of Sangrovit^®^ (3.5 mg/kg *Macleaya cordata* extract) for 28 days in growing/finishing pigs could not demonstrate sanguinarine or chelerythrine residues in organs or tissues, indicating no safety concern when consuming pig-derived meat products [3]. Previous studies have shown that IQ has antimicrobial [1,4], anti-inflammatory [5,6], antioxidant [7] and immunomodulatory effects [8]. In livestock, supplementation with IQ has been shown to increase growth performance in chickens, due to its anti-inflammatory effects [9,10]. In weaning pigs, dietary supplementation with IQ improved growth performance, nutrient digestibility and health status, including anti-inflammatory and antimicrobial beneficial effects [11,12]. In growing pigs, dietary supplementation with IQ decreased the diarrhea score and enhanced the intestinal barrier function [13,14]. Moreover, IQ supplementation was found to reduce the stress response of piglets during transportation by reducing cortisol levels [15]. Until now, there has been a lack of studies on sow dietary supplementation with IQ by which to investigate the effects on sows and piglets.

Farrowing involves high stress levels in sows that cause a reduced feed intake during the perinatal period, which, together with a high demand of energy and nutrients during lactation, can lead to excessive weight loss. Moreover, the sow’s reproductive performance can be reduced, leading to an extended estrus interval and a decreased likelihood to return to estrus [16]. Ultimately, the negative effects of stress can affect piglets’ survival and growth performance [17,18]. A sow’s diet and feed intake not only affect her growth performance but also have a decisive role in the composition of colostrum [19,20], which affects the survival, intestinal development and growth of piglets.

In this study, we hypothesized that stress at farrowing could be decreased by supplementing the diet of sows with IQ. The aim of the study was to investigate if IQ supplementation would decrease stress at farrowing, when supplemented from the last third of the gestation period, or if it would be sufficient from the entrance to the maternity barn one week before farrowing, when feed intake is highly reduced. In addition, the goal was to investigate if supplementation of IQ in a sow’s diet throughout the lactation period would improve colostrum and milk quality, and, consequently, would have a positive outcome on the piglets’ health and performance.

## 2. Materials and Methods

### 2.1. Animals, Diets and Housing

All experimental procedures were in accordance with European and Belgian regulations concerning laboratory animal welfare with protocol number 18-2049, reviewed and approved by the Animal Ethical Committee of the University of Liège.

Sows and piglets were housed at the Walloon Agricultural Research Centre (Gembloux) until weaning. Landrace sows were artificially inseminated with Piétrain semen. From one week after artificial insemination, sows were housed in groups on straw until gestation day 109 (G109). From one week before farrowing until the end of lactation, sows were moved to individual farrowing units that were equipped with wood shavings, a heat lamp and an extra rear space for sows and piglets, accessible by day 5 after delivery (6.8 m^2^).

Twenty-three sows were fed a standard gestation diet until G80, after which they were divided into three dietary groups. The allocation of sows to the different diets considered parity (primiparous vs. multiparous), back-fat thickness and sow performance. The experimental group one (IQ1, *n* = 7) received dietary supplementation with IQ at 90 ppm (i.e., 90 g per ton of feed of the product Sangrovit^®^ Extra (Phytobotics Futterzusatzstoffe GmbH, Eltville, Germany); equivalent to 0.45 ppm of sanguinarine, the active ingredient) from G80 to G107 (at the entrance to the maternity barn) and supplementation with 150 ppm of IQ (equivalent to 0.75 ppm of sanguinarine) from G107 until weaning (on day 21 of lactation (L21). The second experimental group (IQ2, *n* = 8) received a supplementation of 150 ppm of IQ from G107 until weaning (L21), and finally, a control group (NC, *n* = 8) of sows that only received the basal diet (see basal diet composition in Table 1).

At weaning (on L21), 24 female piglets (1 piglet/sow, 8 piglets/treatment) were moved to the Animal Production Centre in Gembloux, where two piglets of the same treatment were housed in one pen (1.5 m^2^); the temperature on arrival day was set at 26 °C. Piglets were fed a standard post-weaning diet devoid of antibiotics, prebiotics, probiotics, and non-starch polysaccharide (NSP) enzymes.

### 2.2. Sows’ Performance Parameters, Blood, Colostrum and Milk Analyses

#### 2.2.1. Sows’ Performance Parameters

The body weight of each sow, back-fat thickness (BFT) and back-muscle thickness (BMT) were recorded at G78 and G106, and lactation days L21 and L28, to determine the changes between periods. BFT and BMT were measured at the last rib at 60 mm from the midline by ultrasound with a linear 5 MHz probe with a Vetko plus (Noveko, Quebec, QC, Canada). Feed intake of the sows was measured by the DAC (Compufeeder^®^, Insentec B.V., Lemelerveld, The Netherlands) system in gestation, and the GESTAL (Gestal FM^®^, Jyga Technologies, Quebec city, QC, Canada) feeding system during lactation. The wean-to-estrus interval was followed for changes before the study, during the study and one round after the study.

The duration of farrowing was determined by recording the time of birth of the first piglet and the time of the expulsion of the placenta. The piglet expulsion rate was calculated by dividing the duration of farrowing by the total number of piglets born. The number of piglets born per sow was also recorded, as well as the average weight per living piglet, per farrowing sow.

The sows’ reproductive performance was also estimated using the number of piglets born alive per sow per year, and the number of piglets weaned per sow per year. These parameters are measured by using the data collected from the three most recent reproduction cycles of each sow. The reproductive parameters were calculated for the period before the experiment, during the experiment and one cycle after the experiment. Not all sows could be followed for all three calculations, due to low parity sows with missing previous data for calculation and high parity sows being taken out from the barns.

#### 2.2.2. Sow’s Blood Analyses

Blood was taken three times from four sows per treatment on G107 before entering the maternity barn, immediately after farrowing, between 4 and 6 h after the first piglet was born, and on day 7 of lactation (L7). All samples were taken at the same time of the day (13:00 p.m.). Blood was collected in an EDTA-vacutainer and centrifuged for 15 min at 2000× *g*. Plasma was stored at −20 °C until analysis.

Glucose was measured in the sows’ plasma by the mutarotase-GOD spectrophotometric method (LabAssayTMGlucosse, Fujifilm, Wako, Japan) according to the manufacturer’s instructions. Insulin and cortisol were measured by ELISA according to the manufacturer’s instructions (Mercodia Porcine Insulin ELISA, Mercodia AB, Uppsala, Sweden; Cortisol Assay, R&D Systems, Inc., Minneapolis, MN, USA, respectively).

#### 2.2.3. Colostrum and Milk Analysis

Colostrum was manually sampled from functional teats within the first 4 h after the birth of the first piglet. Milk samples were collected weekly after an intramuscular injection of 1.5 mL of oxytocin at 10 Un/mL (V.M.D. nv/sa, Arendonk, Belgium). Colostrum and milk samples were stored at −20 °C until analysis, after filtration with sterile medical gauze. Protein and fat contents in colostrum and milk were determined by Fourier transform infrared spectroscopy on a Standard Lactoscope FT-MIR automatic (Delta Instruments, Drachten, Friesland, The Netherlands). The predictive models provided by the manufacturer were adapted for sow’s milk [21].

The immunoglobulin content in colostrum and milk was measured by ELISA using IgG- and IgA-specific anti-pig antibodies (Bethyl Laboratories, Montgomery, Alabama, AL, USA, and R&D Systems, Abingdon, Oxon, UK), following the manufacturer’s recommendations. The plates were read at 450 nm on a 96-well plate reader (Stat-fax 2100, Awareness Technology Inc., Palm City, FL, USA).

### 2.3. Piglets’ Performance Parameters and Diarrhea Score

#### 2.3.1. Piglets’ Performance

Between birth and one day of lactation, the number of piglets per litter was equilibrated with piglets from a treatment-corresponding sow, and, thus, data is provided per lactating sow. Piglets were weighed at birth and once a week until weaning, and litter size was recorded to calculate the mortality rate during the lactation period. On L21, one or two piglets per sow were weaned and transferred to a post-weaning facility. Four days after the weaning day (25 days old), the piglets were sacrificed to determine ileal histology and gene expression in the ileum (in detail in Section 2.4).

#### 2.3.2. Diarrhea Score

During the lactation period, fecal scoring was performed on three consecutive days in the first, second, and third weeks, and once in the last week (L25). This resulted in 10 observation times per litter. The consistency of the feces was evaluated and given a score from 0 to 4 (0 = hard pellet, 1 = soft, dry pellet, 2 = soft, shaped wet pellet, 3 = unshaped soft pellet, 4 = watery diarrhea). This score was given for individual observations, and an average was calculated for the litter. The number of times with the presence of diarrhea recorded (score 4), the diarrhea percentage of the total number of observations as well as the average consistency of feces, which was calculated as the geometrical mean of the scores from all observations, are presented.

### 2.4. Sampling of Piglets’ Intestinal Tissues, Histology and Gene Expression Analysis

#### 2.4.1. Sampling of Intestinal Tissues

Four days after weaning, piglets (8 piglets per treatment, *n* = 24) were euthanized by isoflurane inhalation for anesthesia, followed by bleeding. From the group IQ1 (*n* = 7), two piglets were taken from the same sow so as to have an equal number of piglets per group. Tissue samples of the intestine from the ileum segment were collected and, after rinsing with saline solution, were either snap-frozen in liquid nitrogen or fixed in 4% formalin. Frozen samples were stored at −80 °C until further analysis.

#### 2.4.2. Histology

Formalin-fixed tissue samples were transferred to 70% ethanol and stored until being embedded in paraffin and stained with hematoxylin and eosin, according to standard procedures for histomorphometric analysis. Villus heights and crypt depths were measured on 15–20 well-oriented villus/crypt samples per animal by 10-fold magnification microscopy (Olympus BX51, Olympus, Tokyo, Japan).

#### 2.4.3. Gene Expression Analysis

Total RNA from frozen ileum intestinal tissue was extracted using a ReliaPrep™ RNA Tissue Miniprep System Kit (Promega, Madison, Wisconsin, WI, USA), according to the manufacturer’s instructions. RNA concentration and quality were determined by Nanodrop 2000 (Thermo Fisher Scientific, Wattham, MA, USA) and agarose gel (1%), respectively.

Extracted RNA was converted to single-stranded cDNA using GoScript™ Reverse Transcription Mix (Promega, Madison, Wisconsin, WI, USA), following the manufacturer’s instructions. The analysis of gene expression was done on a StepOne Plus real-time PCR system (Applied Biosystems, Whaltham, MA, USA) using SYBR Premix Ex Taq II (TakaraBio, Shiga, Japan). This included the housekeeping genes glyceraldehyde-3-phosphate dehydrogenase (GAPDH) and beta-actin (ACTB), and the immune-related genes tumor necrosis factor-alpha (TNF-α), interleukin 6 (IL-6) and interleukin 10 (IL-10). Primers and their references are shown in Table 2. QPCR was performed under standard conditions (denaturation at 95 °C for 5 s, annealing at 60 °C for 30 s, and elongation at 72 °C for 30 s), and primer specificity was verified through melting curves. Relative gene expression was calculated by using a standard curve created with a 5-fold dilution series of a pool-sample, created with equal volumes of each individual sample. The geometrical mean of the housekeeping genes was used to normalize gene expression for comparison and statistical analysis of the results.

### 2.5. Statistical Analysis

Sows, or a litter of piglets, were considered as the experimental unit. Data were analyzed by one-way ANOVA, with each treatment group as the main factor, or by mixed-effects analysis (REML), with treatment and time as the main factors and the sow as a random factor. The overall parity was balanced within and between treatments and, therefore, it was not included in the final model, since the main effects of interest were those caused by dietary supplementation with isoquinoline alkaloids. Dunnett’s multiple comparisons test was used to analyze the differences of the supplemented groups IQ1 and IQ2 compared to the control group NC. A *p*-value ≤ 0.05 was considered statistically significant, whereas *p*-values between 0.05 and 0.1 were considered as a tendency. Graphs and tables show mean ± standard deviation (SD). Statistical analysis was performed with GraphPad Prism 8.0 (GraphPad Software, Inc., San Diego, CA, USA).

## 3. Results

**Figure 1:**
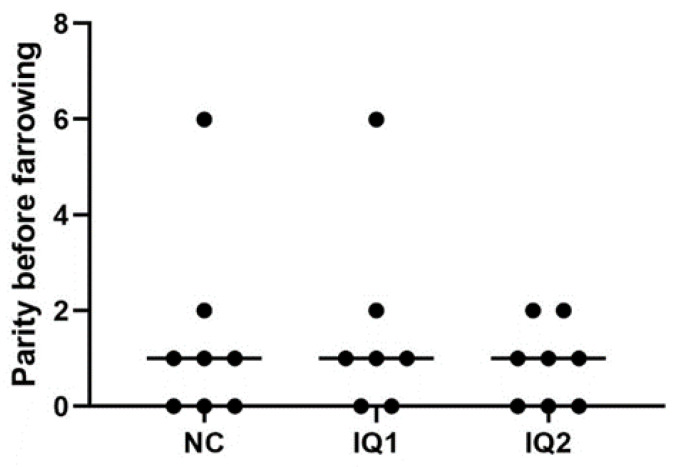
Parity of sows, before the farrowing, included in the experiment. Sows received either a basal diet (NC), or supplementation with isoquinoline alkaloids (IQ) during gestation and lactation (IQ1), or only during the lactation period (IQ2).

It is known that parity can have notable effects, with the biggest differences appearing between gilts and experienced sows. However, due to the characteristics and possibilities available at the experimental farm, homogeneity by parity was not feasible. Instead, sows were allocated to the different treatment groups, considering several factors, including parity (Figure 1).

### 3.1. Sows’ Performance, Blood, Colostrum and Milk Parameters

#### 3.1.1. Sows’ Performance Parameters

No statistically significant differences in body weight were observed between treatments (Table 3). Moreover, time had a significant effect on body weight (*p* < 0.0001), and the interaction between treatment and time showed a statistical tendency (*p* = 0.07). The IQ1 group of sows started with a slightly higher body weight than NC and IQ2, which remained higher at every measurement, even though no statistical significance was found. However, total body weight loss for the time spent in the maternity barn (G106-L28) showed a tendency between IQ2 and NC (*p* = 0.096), whereas the body weight loss of the IQ2 sows was lower than that of NC sows. Even though no statistical relevance was found for IQ1 sows, they had intermediate values of maternity weight loss. There were no differences in feed intake between treatments.

Back-fat and back-muscle thickness were not found to differ between treatments. However, IQ1 showed a tendency for a higher BFT (*p* = 0.067) compared to the control group. Even though it was not statistically significant, IQ1 showed the lowest reduction in back-fat thickness during the time period spent at the maternity barn (*p* = 0.125).

#### 3.1.2. Sows’ Reproductive Performance

The sows’ performance at farrowing was measured as the time between the expulsion of the first piglet and the placenta, and the piglet expulsion rate (Table 4), without statistical differences between treatments. The number of piglets born in total and alive per sow was somewhat lower in IQ2 sows (0.05 < *p* < 0.1), which also showed high variation. Thus, no significant differences were found in the piglets measured by factors such as the number of piglets and mortality rate at birth, neither were differences found in the average weight per piglet at birth.

The reproductive performance of the sows was evaluated during the cycle before the experiment, in the cycle of the experiment, and in the cycle after the experiment (Table 5). During the experiment, the number of piglets born alive per sow was decreased in the IQ2 group compared to the control group (*p* < 0.05). No other differences were observed between dietary treatments. Furthermore, all groups had a decreased number of weaned piglets per sow during the experimental cycle due to the inclusion of the primiparous sows (*p* < 0.05) which disappeared on the following cycle.

#### 3.1.3. Sows’ Blood Parameters

**Figure 2:**
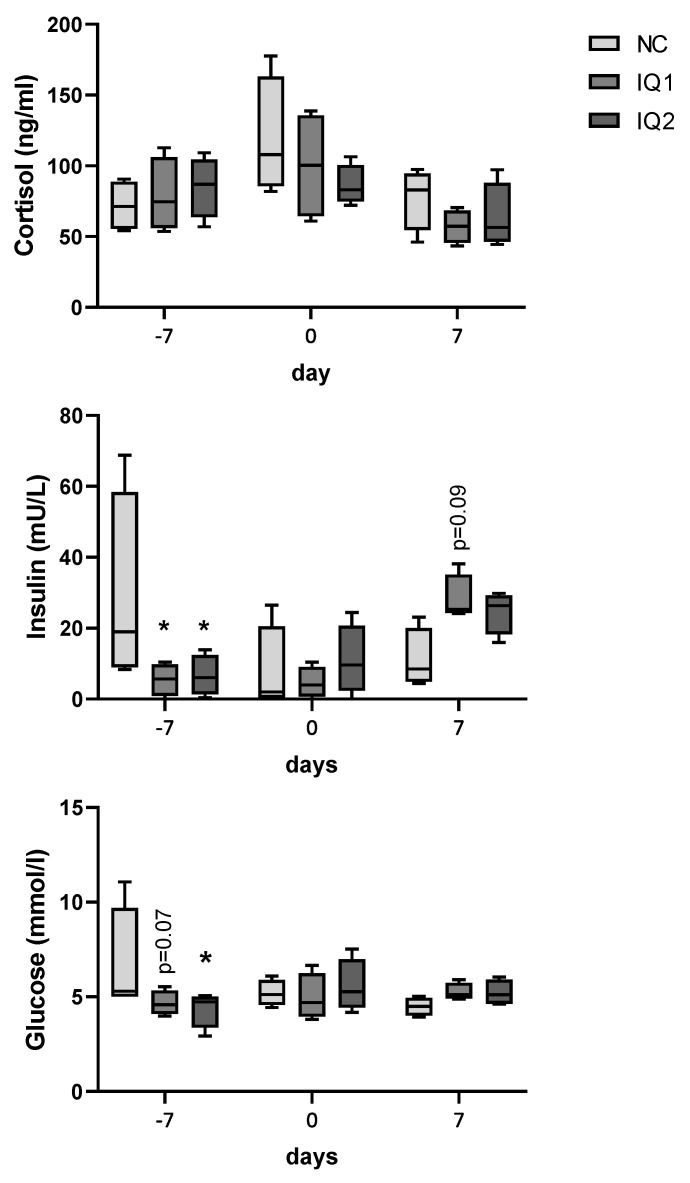
Cortisol, insulin and glucose levels in sows’ plasma one week before farrowing (−7 d), on the farrowing day (0 d), and one week after farrowing (7 d). Sows received either a basal diet (NC), or supplementation with isoquinoline alkaloids (IQ) during gestation and lactation (IQ1), or only during the lactation period (IQ2). Data presented in boxplot: box line + median, mean ± bars (inner fences defined by Tukey’s method). Statistical differences were considered when *p* < 0.05, as indicated by asterisks (*, *p* < 0.05); statistical trends were considered when 0.1 > *p* > 0.05.

Cortisol, glucose and insulin concentrations in plasma were determined (Figure 2). Time and the individual sow were identified as statistically significant factors (*p* < 0.05), due to a high variation between sows. Cortisol levels were not different between groups. Insulin plasma concentration was lower in both the IQ1 and IQ2 groups (*p* < 0.05) compared to the NC group, one week before farrowing, when the IQ2 sows had received the supplementation for one meal (G107). At one week after farrowing, insulin levels tended to be higher in IQ1-treated sows compared to NC sows (0.5 < *p* < 0.1). For the NC group, one sow had rather high insulin values on the three moments of samplings, although a statistical test to identify outliers did not consider it as an outlier. Glucose concentrations were lower one week before farrowing, after one meal with the supplement for IQ2, in both treatment groups compared to controls (NC) (IQ1, 0.5 < *p* < 0.1; IQ2, *p* < 0.05).

#### 3.1.4. Colostrum and Milk Composition

**Figure 3:**
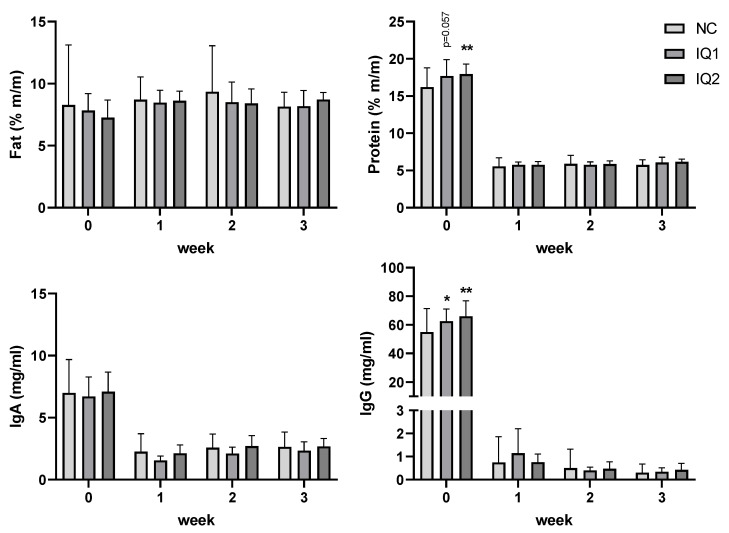
Fat and protein (%, top graphs), immunoglobulin A and immunoglobulin G (mg/mL; lower graphs) content in colostrum and milk. Sows received either a basal diet (NC), or supplementation with isoquinoline alkaloids (IQ) during gestation (90 ppm) and lactation (150 ppm) (IQ1), or only during the lactation period (IQ2). Milk collection was performed once a week from farrowing (week 0) until day 21 of lactation (3rd week). Data presented as mean ± SD. Statistical differences were considered when *p* < 0.05, as indicated with * (*, *p* < 0.05; **, *p* < 0.01), statistical trends were considered when 0.1 > *p* > 0.05.

Colostrum (collected a max. of 4 h after the birth of the first piglet) and milk (L7, L14, L21) were collected from all sows (see Figure 3). Fat content (%) increased between farrowing (colostrum) and L7, remaining stable thereafter (Figure 3, top left). No treatment effect was observed concerning the fat content of milk or colostrum.

The protein content (%) in colostrum was higher in IQ1 and IQ2 groups than in the NC group (0.5 < *p* < 0.1; and *p* = 0.008, respectively). This effect was not observed in the weekly collected milk samples from one week after farrowing (Figure 3, top right).

In the colostrum, high levels of IgA and IgG were observed in all groups, which decreased thereafter in the milk. IgA colostrum and milk levels did not show any differences due to sow supplementation with IQ. IgG concentration in colostrum was higher in IQ1 and IQ2 groups compared to the control group (*p* = 0.050 and *p* = 0.002, respectively) (Figure 3, bottom right).

### 3.2. Piglets’ Performance Parameters and Diarrhea Score

#### 3.2.1. Piglets’ Performance Parameters

Pigs were weighed weekly (Table 6). At birth, the weight of piglets delivered by sows in the different dietary groups showed no differences, with an overall average birth weight of 1.47 ± 0.21 kg per piglet. No differences were observed in average weight or average daily weight gain per piglet in the experimental groups, compared to controls.

#### 3.2.2. Diarrhea Score of Piglets

Regarding the overall fecal observations and diarrhea scores, no significant differences were observed between treatments (Table 7) as to the presence of diarrhea or average consistency. Nonetheless, piglets in the IQ1 group showed a higher, but not significant, percentage of diarrhea occurrence compared to NC.

### 3.3. Piglets’ Ileum Gene Expression and Histomorphometry

From each piglet that was euthanized (*n* = 8 per treatment), an ileal section was analyzed to determine the villus height and crypt depth of 15–20 well-oriented villi and crypts. The average value per piglet was used to analyze the treatment effect. No treatment effect was observed concerning villus height, crypt depth, or their ratios (Table 8).

**Figure 4:**
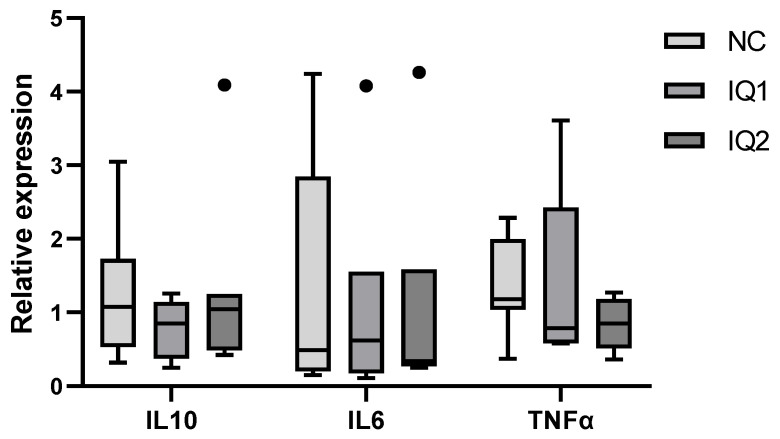
Relative gene expression in ileal tissue of piglets 5 days after weaning on day 21, born from sows that received either a basal diet (NC), or supplementation with isoquinoline alkaloids (IQ) during gestation (90 ppm) and lactation (150 ppm) (IQ1), or only during the lactation period (IQ2). ACTB and GADPH were used as reference genes. A standard dilution curve was used to calculate the relative gene expression of the target genes, using the geometric average of the housekeeping genes (*n* = 7/group). Data presented in boxplot: box line + median, mean ± bars (inner fences) and Tukey’s outliers (circles). Statistical differences were considered when *p* < 0.05.

Due to abnormally low values in the housekeeping genes, one individual from each group was excluded from the intestinal gene expression analysis. Nonetheless, the gene expression of IL-10, IL-6 and TNF-α in the ileum showed high variation, as seen in Figure 4.

## 4. Discussion

Feed intake and metabolic state during late gestation and lactation periods can have major effects not only on maternal body condition but also on piglet health, as well as on the subsequent reproductive performance [27]. Excessive weight loss during lactation can increase the weaning-to-estrus interval [28,29] and thus, decrease the rates of ovulation and conception [30,31]. In our study, IQ2 sows showed a tendency to decrease the total body weight loss compared with NC sows, and IQ1 sows had intermediate values. At the same time, the feed intake from G107 to weaning showed no differences between the three groups, which indicates that the dietary supplement with IQ had a positive effect on sows’ feed efficiency. Goodarzi Boroojeni et al. also found that post-weaning piglets fed a diet supplemented with a preparation of IQ at 120 mg/kg displayed an improvement in apparent nutrient digestibility and feed conversion ratio, compared with those fed a control diet [12].

Back-fat thickness (BFT) is considered an objective indicator of the total fat content of the sows’ body composition and body condition [32,33]. Kim et al. observed that the sows with 17–21 mm BFT on day 106 of gestation had larger litter sizes [34]. Moreover, it has been shown that an excessive loss of back fat during lactation increased the weaning-to-estrus interval, as well as decreasing the pregnancy rate and the productive lifetime of sows [35,36]. In our study, BFT at G106 of sows in the three groups were all within the expected range. The smallest reduction in BFT was observed in IQ1 sows from G106 to L28. IQ1 sows also showed a tendency for higher BFT compared with the control group on L28, while IQ2 sows showed intermediate values. Therefore, this indicates that diets with IQ had a positive effect on maternal body condition. On the other hand, the reproductive performance parameters (at farrowing and the performance of piglets in the lactation period) were unaffected by the IQ treatment. Despite the lower number of piglets born alive per sow per year in the IQ2 group compared to the control group, this reduction was not observed in the IQ1 group. Thus, IQ supplementation would not seem to be the cause, especially since sows in the IQ1 group had received IQ supplementation for a longer period than those in IQ2 (only since G107), and the fact that no other parameters measured at farrowing (i.e., piglets born alive, the total number of piglets, and survival during lactation) showed differences between treatments. Thus, it would seem more of a coincidence that could be related to the parity of the sows.

Cortisol is an indicator of stress in swine, which is produced by the adrenal gland in response to increased anxiety, caused by various stressors [37,38]. In our study, cortisol levels increased at farrowing compared to the previous sampling point one week before, but IQ diets did not reduce the cortisol concentration in plasma. In the same area, Le et al. also found that a diet supplemented with 0.15 g/kg IQ fed to growing pigs had no effects on salivary cortisol concentrations during heat stress [14]. Glucose is the most important nutrient for the sows’ mammary gland metabolism [39]. The mammary glands use a lot of the available plasma glucose to synthesize milk [40]. Cortisol levels increase during stress, increasing glucose and, subsequently, insulin concentrations. However, as the cortisol level was unaltered, it is difficult to link lower glucose and insulin concentrations to lower stress. Seven days after farrowing, glucose and insulin concentrations were numerically higher in both IQ groups, again without any trend showing differences in cortisol between treatments.

It is known that in the first week after farrowing, blood glucose levels increase and then decrease afterward, while blood insulin concentrations increase during the first two weeks post-farrowing [39]. As insulin stimulates glycogen storage and lipid and protein synthesis, and inhibits the breakdown of these molecules, the numerically higher insulin concentration in the IQ groups may be related to the lower body weight loss over the whole period for the IQ2 group, and the higher BFT at L28 for the IQ1 group. Interestingly, these blood parameters were similar for both IQ groups, suggesting that IQ supplementation had immediate effects, from the first meal after arrival at the maternity barn. Hence, IQ supplementation during the maternity period may be sufficient to influence glucose metabolism in sows. Moreover, isoquinoline alkaloids, such as sanguinarine, have long been studied for their antidiabetic properties [41], through glucose-lowering effects due to their capacity to inhibit α-glucosidase activity in the small intestine, and a decrease in intestinal glucose absorption [42]. In sows, as well as in other species, it has been shown that insulin resistance appears during late gestation, as an adaptation to the high glucose demands of pregnancy [43]. Furthermore, sanguinarine has been shown to inhibit digestive enzyme activities (amylase, lipase and protease) and their relative expression in the digestive tract of Lepidoptera larvae [44]. Thus, sanguinarine and other isoquinoline alkaloids may have potential as anti-diabetic molecules and could have effects on pancreatic function, but further research is needed.

Piglets are born with low body energy stores and a lack of prenatal transfer of maternal immunoglobulins. Therefore, newborn piglets must receive passive immunity, especially immunoglobulin G (IgG), during the postnatal period from the colostrum and milk in order to get immune protection from pathogens during early life [45,46]. Colostrum is highly digestible and nutritious, and it contains high amounts of proteins, immunoglobulins, and a variety of bioactive components that can promote the development of the intestine, protein synthesis in the muscle, and provide protection against infections for the piglets [47,48]. Moreover, the energy in the colostrum for thermoregulation is efficiently retained [49]. Furthermore, an increase in the immunoglobulin content in colostrum, as seen in our study, can provide a higher level of (passive) immunity to piglets in early life, which is extremely important given the fact that piglets born from hyper-prolific sows have reduced access to suckling, and the fact that the piglets’ own immune system is not yet fully developed [50] at early weaning at 3–4 weeks of age. In addition, isoquinoline alkaloids such as sanguinarine have been shown to have immunomodulatory effects, due to the inhibition of the NF-kB inflammatory pathway [8]. Consequently, lower levels of systemic inflammation in IQ-supplemented sows could have contributed to the increase in IgG content in colostrum.

In our study, both IQ groups had a positive effect on colostrum protein and IgG content, which is an important finding. Chen et al. found that diets supplemented with a preparation of IQs fed to weaned piglets significantly increased the amount of serum IgG compared with the control group [7]. Liu et al. also found that serum IgG concentration was enhanced in the blood of growing pigs fed with *Macleaya cordata* extract-supplemented diets [13]. As mentioned before, the higher IgG content in colostrum might have provided the piglets with a better passive immunity, which is important during the first weeks of life, before their own immune system is mature. Due to this beneficial effect, theoretically, we may have expected a better growth performance for the piglets of the IQ groups. However, no effects due to maternal treatment were observed on piglets’ growth performance or diarrhea, nor on the intestinal absorptive capacity and the inflammatory response (IL6, IL10, TNF-α) four days after weaning. Even though positive effects on the progeny were not observed, it seemed that the piglets responded quite differently to weaning stress, as seen by the high variation in gene expression, which may further complicate the visualization of differences between treatments. Interestingly, previous studies showed that diets supplemented with IQ or *Macleaya cordata* extract increased BW, ADG and feed intake, and reduced diarrhea in weaned piglets [7,11]. Other research also indicated that a diet supplemented with *Macleaya cordata* extract fed to weaned piglets significantly increased villus height, and the ratio of villus height to crypt depth in the duodenum, jejunum and ileum, and decreased the crypt depth in the jejunum [51]. Furthermore, IQ supplementation inhibited the expression of IL-6 in the ileal tissue of broiler chickens [10]. It should be taken into account that the number of sows and piglets included in this study is rather low, and the response to IQ supplementation might have been affected by the high sanitary conditions of the experimental farm.

## 5. Conclusions

IQ dietary supplementation to sows had its main effect on the sows’ metabolisms, reducing body weight loss during lactation. IQ improved colostrum quality, increasing the protein and IgG content, possibly providing piglets with better passive immunity. Hence, it seems that IQ supplementation to sows had its main effects on the sows directly, but the effects on the progeny should be further investigated. Providing IQ to sows from the time of entrance to the maternity barn might be sufficient to induce these effects.

## Figures and Tables

**Table 1:** Calculated composition of sows’ basal diet during the gestation and the lactation periods.

Ingredient (%)	Gestation	Lactation
Wheat	20.83	21.81
Barley	18.70	14.00
Wheat bran	12.50	10.00
Corn	12.00	12.50
Beet pulp	7.40	4.90
Soybean hulls GMO	4.00	3.00
Palm oil	4.00	3.00
Rice bran	3.50	3.50
Nutex 68 (Dumoulin Inc.) *	3.40	3.40
Corn DDGS	2.80	2.00
Sunflower meal	2.70	-
Soybean meal GMO	-	9.60
Rapeseed meal	-	1.20
Molasses	2.00	2.00
Cocoa hulls	2.00	2.00
Corn gluten	1.50	1.50
Limestone	0.98	1.53
Animal fat	0.18	2.17
Salt	0.25	0.51
L-Lysine 50%	0.43	0.47
Na-Bicarbonate	0.38	-
Monocalcium-phosphate	0.05	0.42
Premix (oligo, vitamin, enzymes)	0.30	0.30
L-Threonine	0.06	0.03
Vitamin E	-	0.02
L-Tryptophan	-	0.01
Threonine + Methionine 70/30	0.05	0.12
Total	100.00	100.00
Isoquinoline alkaloids (ppm)	90	150
Dry matter ^a^	874	876
Crude protein ^a^	130	155
Crude fat ^a^	47	65
Crude fiber ^a^	78	64
Crude ash ^a^	57	65
Starch ^a^	342	321
Total Sugar ^a^	47.95	51.90
Calcium ^a^	7.00	9.76
Phosphor-d ^a^	2.43	3.15
Ca/P ^a^	1.36	1.68
Sodium ^a^	2.40	2.38
Non-starch polysaccharide ^a^	264	230
Linoleic acid (C 18:2) ^a^	13.33	14.61
Linolenic acid (C 18:3) ^a^	5.35	5.67
Lysine-d ^a^	5.05	7.26
Methionine-d ^a^	1.77	2.25
Methionine + Cysteine-d ^a^	3.33	4.09
Threonine-d ^a^	3.43	4.79
Tryptophan-d ^a^	1.01	1.45
Net Energy (kcal)	2110	2230
Dietary electrolyte balance (Na+K-Cl, mEq)	220.40	201.70

^a^ given in units of g/kg feed; * Nutex: 10% wheat, 20% wheat bran, 70% linseed; (-d) amino acids given as digestible (apparent ileal digestibility) amounts.

**Table 2:** Primers used for gene expression analysis.

Target Gene	Code		Primer Sequence (5′→3′)	Reference	Accession Number
Actin–beta	ACTB	F	GGA-CTT-CGA-GCA-GGA-GAT-GG	[22]	XM_021086047
		R	GCA-CCG-TGT-TGG-CGT-AGA-GG		
Glyceraldehyde-3- phosphate dehydrogenase	GAPDH	F	CAT-CCA-TGA-CAA-CTT-CGG-CA	[23]	NM_001206359.1
		R	GCA-TGG-ACT-GTG-GTC-ATG-AGT-C		
Tumor necrosis factor-alpha	TNF-α	F	ACT-GCA-CTT-CGA-GGT-TAT-CGG	[24]	NM_214022.1
		R	GGC-GAC-GGG-CTT-ATC-TGA		
Interleukin–6	IL-6	F	AGA-CAA-AGC-CAC-CAC-CCC-TAA	[25]	NM_214399
		R	CTC-GTT-CTG-TGA-CTG-CAG-CTT-ATC		
Interleukin–10	IL-10	F	CTG-CCT-CCC-ACT-TTC-TCT-TG	[26]	NM_214041
		R	TCA-AAG-GGG-CTC-CCT-AGT-TT		

**Table 3:** Sows’ performance data (mean ± SD). Initial body weight, bodyweight change, feed intake, back-fat and back-muscle thickness were measured at the indicated days of experiments during the gestation period (G) and during lactation (L).

Iterms	NC (*n* = 8)	IQ1 (*n* = 7)		IQ2 (*n* = 8)	
Bodyweight (kg)	mean ± SD	mean ± SD	*p*-value	mean ± SD	*p*-value
G76	215 ± 28	233 ± 31	0.407	211 ± 21	0.934
G106	230 ± 21	250 ± 37	0.341	226 ± 17	0.931
L21	202 ± 33	228 ± 43	0.164	210 ± 21	0.796
L28	187 ± 32	215 ± 43	0.114	197 ± 24	0.728
Bodyweight change (ΔKg)					
Gestation (G76–G106)	8 ± 2	9 ± 2	0.954	8 ± 2	0.999
Maternity (G106–L28)	−43 ± 14	−34 ± 13	0.333	−29 ± 14	**0.096**
Feed intake (kg)					
Gestation (G80–G106)	71 ± 4	74 ± 11	0.959	71 ± 3	0.999
Maternity (G106–L28)	174 ± 24	179 ± 29	0.893	175 ± 16	0.994
Back-fat thickness (mm)					
G76	21.1 ± 4.9	22.3 ± 2.7	0.803	21.0 ± 3.9	0.997
G106	20.3 ± 5.2	21.9 ± 4.1	0.663	21.3 ± 4.3	0.839
L28	15.3 ± 3.6	19.7 ± 4.2	**0.067**	17.5 ± 2.6	0.436
Back-fat thickness change (Δmm)					
Gestation (G76–G106)	−0.9 ± 0.6	−0.4 ± 0.8	0.941	0.3 ± 1.1	0.672
Maternity (G106–L28)	−5.0 ± 1.2	−2.1 ± 1.2	0.125	−3.8 ± 1.3	0.615
Back-muscle thickness (mm)					
G76	48.1 ± 6.2	52.4 ± 5.0	0.257	47.5 ± 5.0	0.966
G106	43.5 ± 6.6	46.9 ± 5.7	0.423	45.4 ± 4.1	0.738
L28	40.5 ± 7.2	44.9 ± 5.1	0.249	40.0 ± 5.8	0.978
Back-muscle thickness change (Δmm)					
Gestation (G76–G106)	−4.6 ± 2.1	−5.6 ± 1.6	0.941	−2.1 ± 1.8	0.647
Maternity (G106–L28)	−3.0 ± 8.8	−2.0 ± 5.3	0.934	−5.4 ± 6.9	0.674

Significance was considered when *p* < 0.05, and tendency was considered when 0.05 < *p* < 0.1); this is indicated in bold.

**Table 4:** Sows’ performance at farrowing during the experiment. Sows received either a basal diet (NC), or supplementation with isoquinoline alkaloids (IQ) during gestation and lactation (IQ1), or only during the lactation period (IQ2).

Iterms	NC (*n* = 8)	IQ1 (*n* = 7)		IQ2 (*n* = 8)	
	Mean ± SD	Mean ± SD	*p*-Value	Mean ± SD	*p*-Value
Duration of farrowing (h) *	3.0 ± 0.8	3.3 ± 1.0	0.926	3.4 ± 1.1	0.706
Piglet expulsion rate (piglets/h) *	5.4 ± 1.6	4.4 ± 0.7	0.580	4.3 ± 1.8	0.362
No piglets born alive	15.4 ± 3.6	13 ± 1.7	0.263	12.1 ± 3.5	**0.096**
No piglets born dead	0.6 ± 1.1	1.3 ± 1.7	0.573	0.5 ± 1.4	0.977
No piglets born total	16.0 ± 4.2	14.3 ± 1.8	0.555	12.6 ± 3.9	0.125
Mortality birth rate (%)	3.2 ± 4.9	8.4 ± 11.2	0.408	2.9 ± 8.3	0.998
Weight living piglets at birth (kg/piglet)	1.49 ± 0.16	1.44 ± 0.26	0.942	1.45 ± 0.16	0.989
Total litter weight at birth (kg) (Σ living pigs/lactating sow)	19.91 ± 2.06	18.44 ± 2.73	0.842	17.22 ± 3.29	0.909

* Farrowing duration and piglet expulsion rate NC (*n* = 7), IQ1 (*n* = 3) and IQ2 (*n* = 7). Statistical significance was considered when *p* < 0.05, and a tendency was considered when 0.05 < *p* < 0.1; this is indicated in bold.

**Table 5:** Sows’ reproductive performance during the cycle prior to the experiment, during the experiment, and the cycle after the experiment was recorded. Sows received either a basal diet (NC), or supplementation with isoquinoline alkaloids (IQ) during gestation and lactation (IQ1), or only during the lactation period (IQ2).

Iterms		NC		IQ1			IQ2		
Number of Piglets		Mean ± SD	*n*	Mean ± SD	*n*	*p*-Value	Mean ± SD	*n*	*p*-Value
Previous cycle	Born alive/sow/year	36.2 ± 6.6	5	35.3 ± 7.5	5	0.860	32.7 ± 4.9	5	0.308
	Weaned piglets/sow/year	^a^ 30.9 ± 3.8	5	^a^ 29.3 ± 5.3	5	0.708	^a^ 30.9 ± 2.9	5	0.968
Experiment cycle	Born alive/sow/year	36.1 ± 5.8	8	33.0 ± 5.0	7	0.397	30.0 ± 7.1	8	**0.033**
	Weaned piglets/sow/year	^b^ 28.9 ± 3.1	8	^b^ 26.1 ± 4.1	7	0.455	^b^ 26.3 ± 4.7	8	0.471
Next cycle	Born alive/sow/year	33.6 ± 2.7	4	34.8 ± 2.5	3	0.927	35.8 ± 4.2	3	0.997
	Weaned piglets/sow/year	^ab^ 30.5 ± 1.6	4	^ab^ 25.4 ± 3.6	3	0.302	^ab^ 29.4 ± 2.4	3	0.543

*p*-value indicates differences within each parameter between the control group (NC) compared to IQ1, or NC compared to IQ2. ^a,b^ Different letters indicate differences within the same group over time. Statistical significance was considered when *p* < 0.05, as indicated in bold.

**Table 6:** Performance of piglets born from sows that received either a basal diet (NC) or supplementation with isoquinoline alkaloids (IQ) during gestation (90 ppm) and lactation (150 ppm) (IQ1), or only during the lactation period (IQ2): body weight at birth (kg), on postnatal day 7 (L7), 14 (L14) and 21 (L21, weaning); average daily weight gain (ADWG) at one-week intervals and in the lactation period overall.

Iterms	NC (*n* = 8)	IQ1 (*n* = 7)		IQ2 (*n* = 8) *	
Bodyweight (kg/piglet)	mean ± SD	mean ± SD	*p*-value	mean ± SD	*p*-value
Birth (per lactating sow) *	1.46 ± 0.15	1.42 ± 0.20	0.951	1.43 ± 0.17	0.996
L7	2.69 ± 0.45	2.55 ± 0.35	0.898	2.66 ± 0.35	0.994
L14	4.09 ± 0.57	4.07 ± 0.56	0.998	4.23 ± 0.76	0.886
L21	5.97 ± 0.67	5.62 ± 0.59	0.527	6.13 ± 1.000	0.865
ADWG (Δkg/day/piglet)					
Farrowing–L7 *	0.16 ± 0.05	0.17 ± 0.03	0.953	0.17 ± 0.05	0.868
L7–L14	0.20 ± 0.04	0.18± 0.09	0.778	0.22 ± 0.07	0.621
L14–L21	0.27 ± 0.05	0.21 ± 0.04	0.105	0.26 ± 0.06	0.959
Lactation (Farrowing–L21) *	0.21 ± 0.03	0.20 ± 0.02	0.989	0.22 ± 0.05	0.657
Litter size at birth/lactating sow	13.8 ± 1.7	13 ± 0.6		12.1 ± 2.1	
Litter size P7/lactating sow	12.1 ± 1.4	10.9 ± 1.1		10.8 ± 2	
No piglets P14	11.6 ± 2.3	10.6 ± 1.1		10.3 ± 2.2	
No piglets P21	11.4 ± 2.3	10.1 ± 1.3		9.8 ± 2.2	
No weaned piglets	10.4 ± 2.3	8.9 ± 1.3		8.8 ± 2.2	
Mortality rate lactation period	15.2 ± 21.5	21.8 ± 10.9		18.4 ± 16.4	

No significant differences were found for these parameters between treatments; * *n* = 5 for the IQ1 treatment.

**Table 7:** Diarrhea scores over 10 observations during the lactation period of piglets born from sows that received either a basal diet (NC), or supplementation with isoquinoline alkaloids (IQ) during gestation and lactation (IQ1), or only during the lactation period (IQ2).

Iterms	NC (*n* = 8)	IQ1 (*n* = 7)		IQ2 (*n* = 8)	
	Mean ± SD	Mean ± SD	*p*-Value	Mean ± SD	*p*-Value
Diarrhea score	1.50 ± 1.51	2.29 ± 1.89	0.984	1.75 ± 1.83	0.998
Presence of diarrhea (%)	16.65 ± 16.78	25.39 ± 21.01	0.174	19.44 ± 20.37	0.806
Total number of observations	2.38 ± 2.45	7.00 ± 7.09	0.585	5.13 ± 5.91	0.811
Average fecal consistency	1.86 ± 0.75	2.04 ± 0.57	0.999	2.19 ± 0.55	0.997

**Table 8:** Histomorphological measurements in ileal tissue of piglets 5 days after weaning at day 21, born from sows that received either a basal diet (NC), or supplementation with isoquinoline alkaloids (IQ) during gestation and lactation (IQ1), or only during the lactation period (IQ2).

Histomorphology	NC (*n* = 8)	IQ1 (*n* = 8)		IQ2 (*n* = 8)	
	Mean ± SD	Mean ± SD	*p*-Value	Mean ± SD	*p*-Value
VILLI Height (VH; µm)	323 ± 42	331 ± 25	0.835	334 ± 48	0.688
CRYPT Depth (CD; µm)	170 ± 19	164 ± 19	0.900	164 ± 11	0.897
VH/CD ratio	1.90 ± 0.23	2.03 ± 0.20	>0.999	2.04 ± 0.31	>0.999

## Data Availability

Data obtained is available upon request to the corresponding author.
